# Supplementary material for: High rates of plasmid cotransformation in E. coli overturn the clonality myth and reveal colony development
Source: Sci Rep. 2022 Jul 7;12:11515. doi: 10.1038/s41598-022-14598-9 (PMC9262894; doi:10.1038/s41598-022-14598-9)
Supplement: Supplementary file 7 — Supplementary Information 2. [file 41598_2022_14598_MOESM7_ESM.pdf]

Supplemental information inventory:

Supplemental Figures and Legends:
S1 (related to Figure 1 and 2): Three colony mixture versus a triple fluorescent colony.
S2 (related to Table 1): Colony screening array.
S3 (related to Table 1): Screening colony array using confocal microscopy.
S4 (related to Table 1): Cotransformations using kanamycin resistance.
S5 (related to Figure 5 and Table 1): Triple fluorescent mixture colonies.
S6 (related to Figure 6): Triple fluorescent mixture colonies: 10hr & 17hr.
S7 (related to Figure 7): 20 minute time course for mixture colony #1.
S8 (related to Figure 7): 20 minute time course for mixture colony #2.
S9 (related to Figure 4): Colormetric screening assay for inserts

Supplemental Table:
S1 (related to Table 1): Calculated cotransformation rates with probabilities matrix.
S2 (related to Table 1): Cotransformation rates under new conditions.

Supplemental Movies:
S1A (related to Figure 7): Split fluorescence movie of time course for mixture colony #1.
S1B (related to Figure 7): fluorescence movie of time course for mixture colony #1.
S2 (related to Figure 7 and S7): Split fluorescence movie of time course for mixture colony #2.
S3 (related to Figure 5 and S5): Z-stack projection of a triple fluorescent colony.
S4 (related to Figure 5 and S5): Z-stack projection of a second triple fluorescent colony.

Supplemental Data File
S1 (related to Figure 4, 5, 6, 7, S2, S3, S4 and Table S1): Raw data for cotransformation analysis.
S2 (related to Table S1): Calculated probabilities matrix.

Figure S1

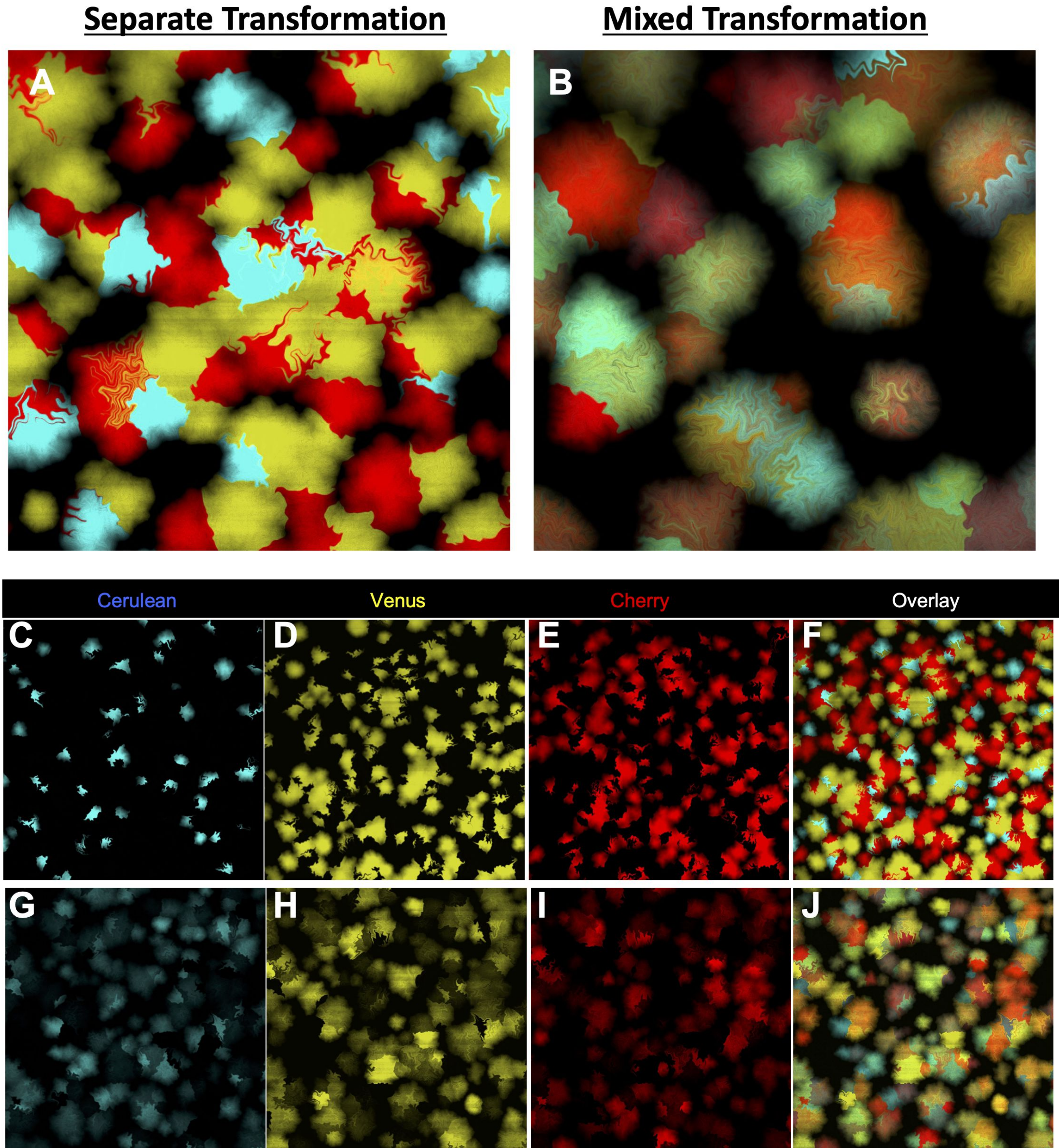
Separate Transformation
Mixed Transformation
A
B
Cerulean
Venus
Cherry
Overlay
C
D
E
F
G
H
I
J

Figure S2

Colony screening array

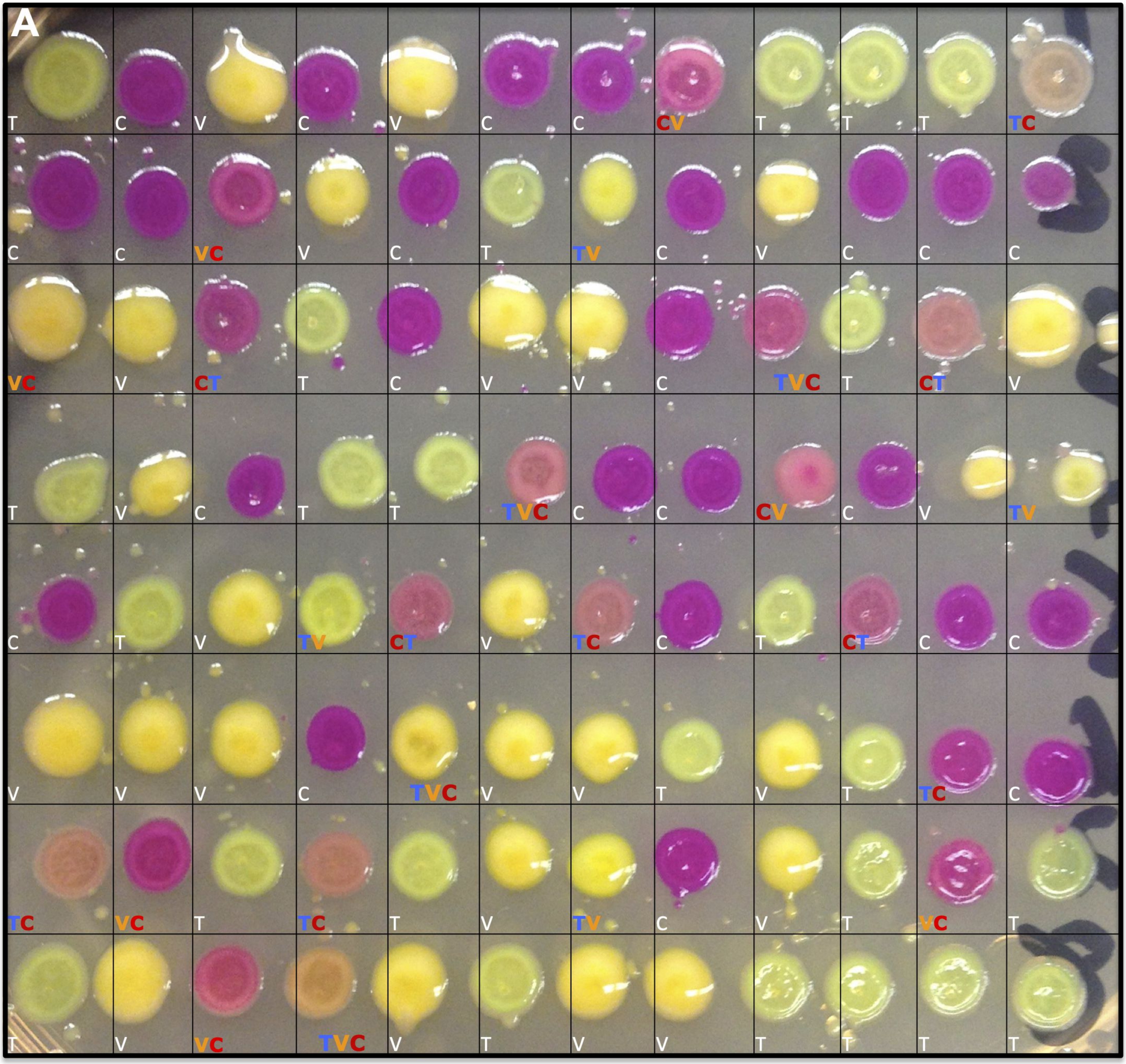

Figure S3

Array screening using confocal microscopy

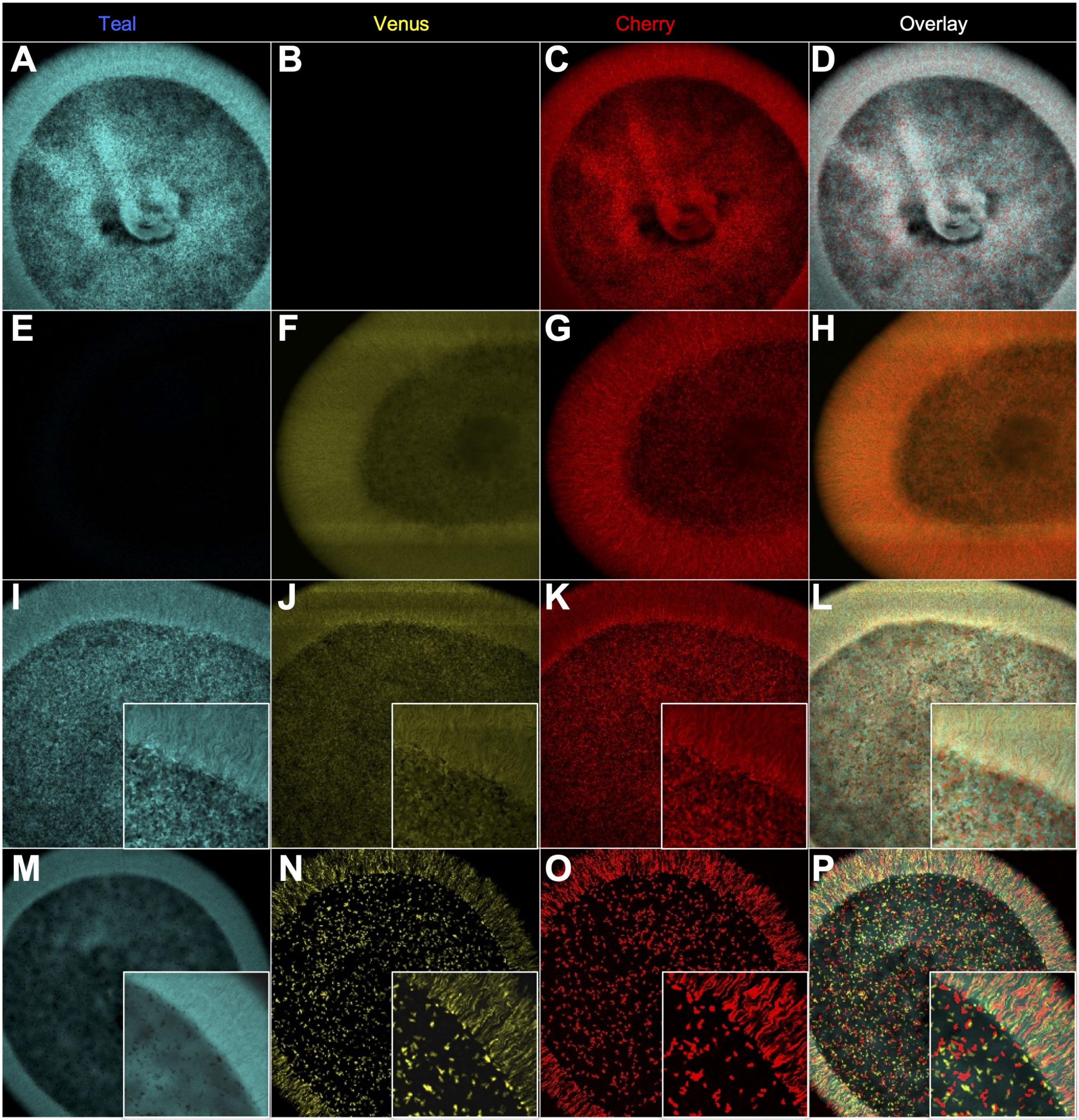

Figure S4

Cotransformations using kanamycin resistance

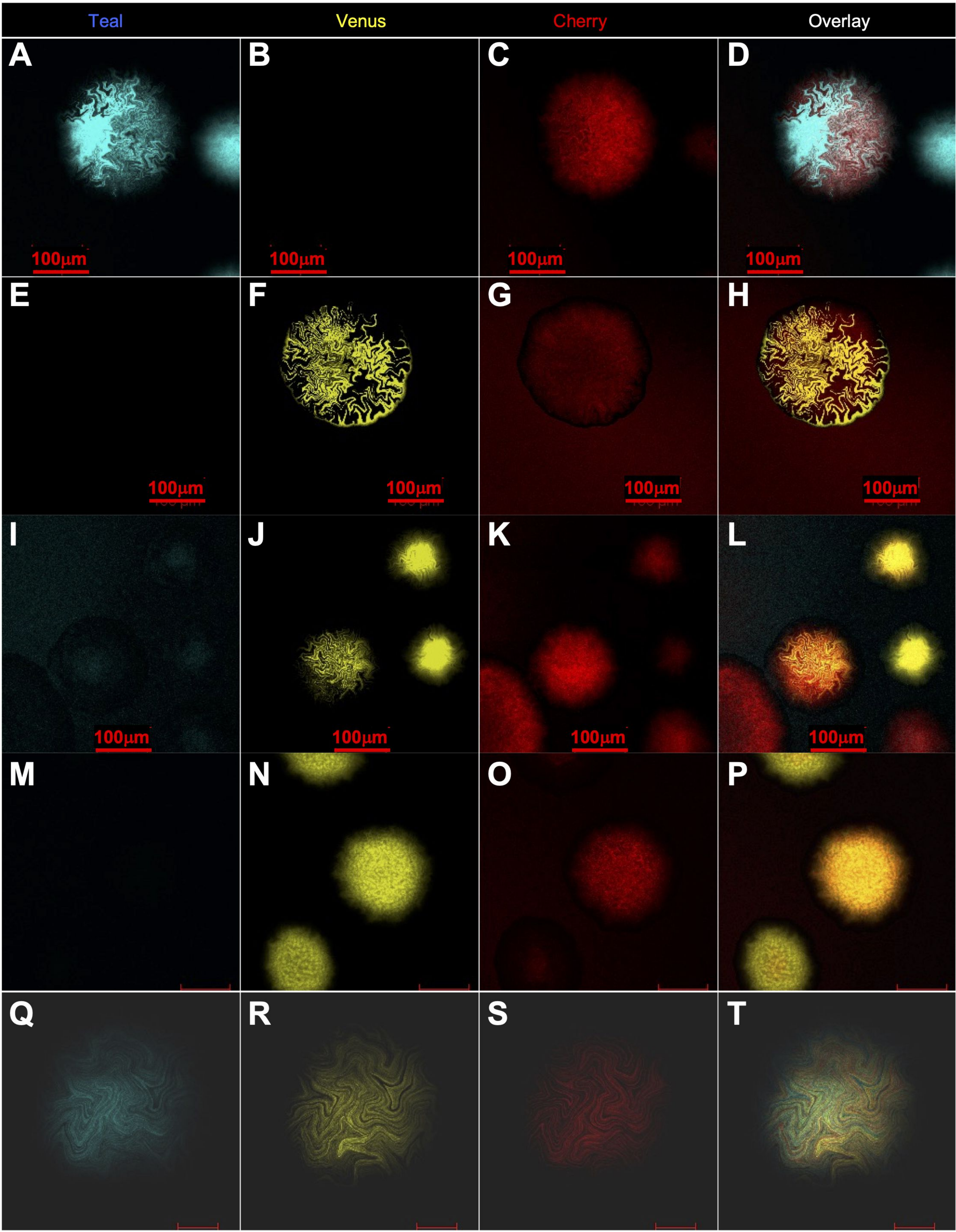

Figure S5

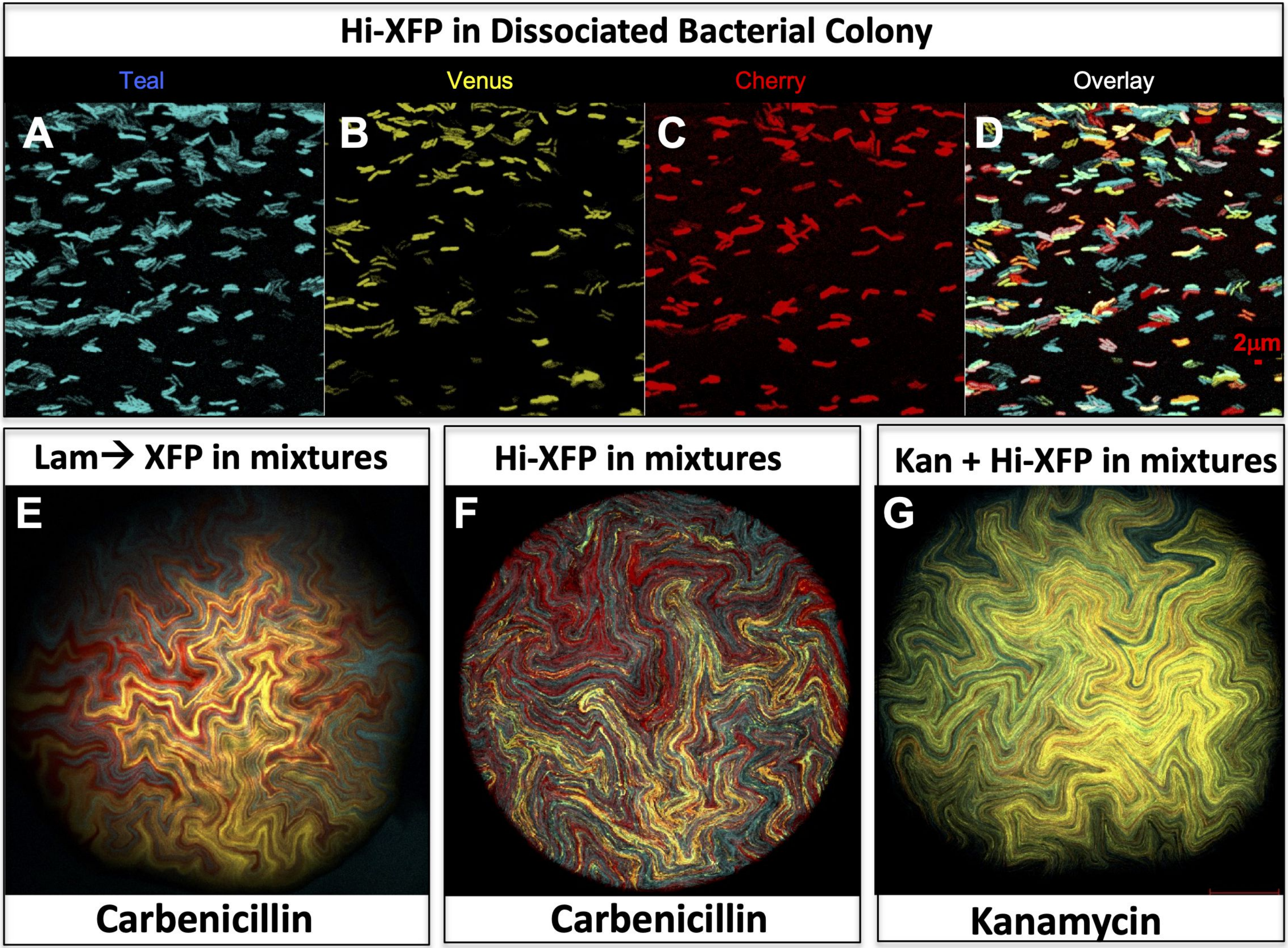
Hi-XFP in Dissociated Bacterial Colony
Teal
Venus
Cherry
Overlay
A
B
C
D
2μm
Lam→ XFP in mixtures
Hi-XFP in mixtures
Kan + Hi-XFP in mixtures
E
F
G
Carbenicillin
Carbenicillin
Kanamycin

Figure S6

Triple flourescent mixture colonies 10hrs and 17hr

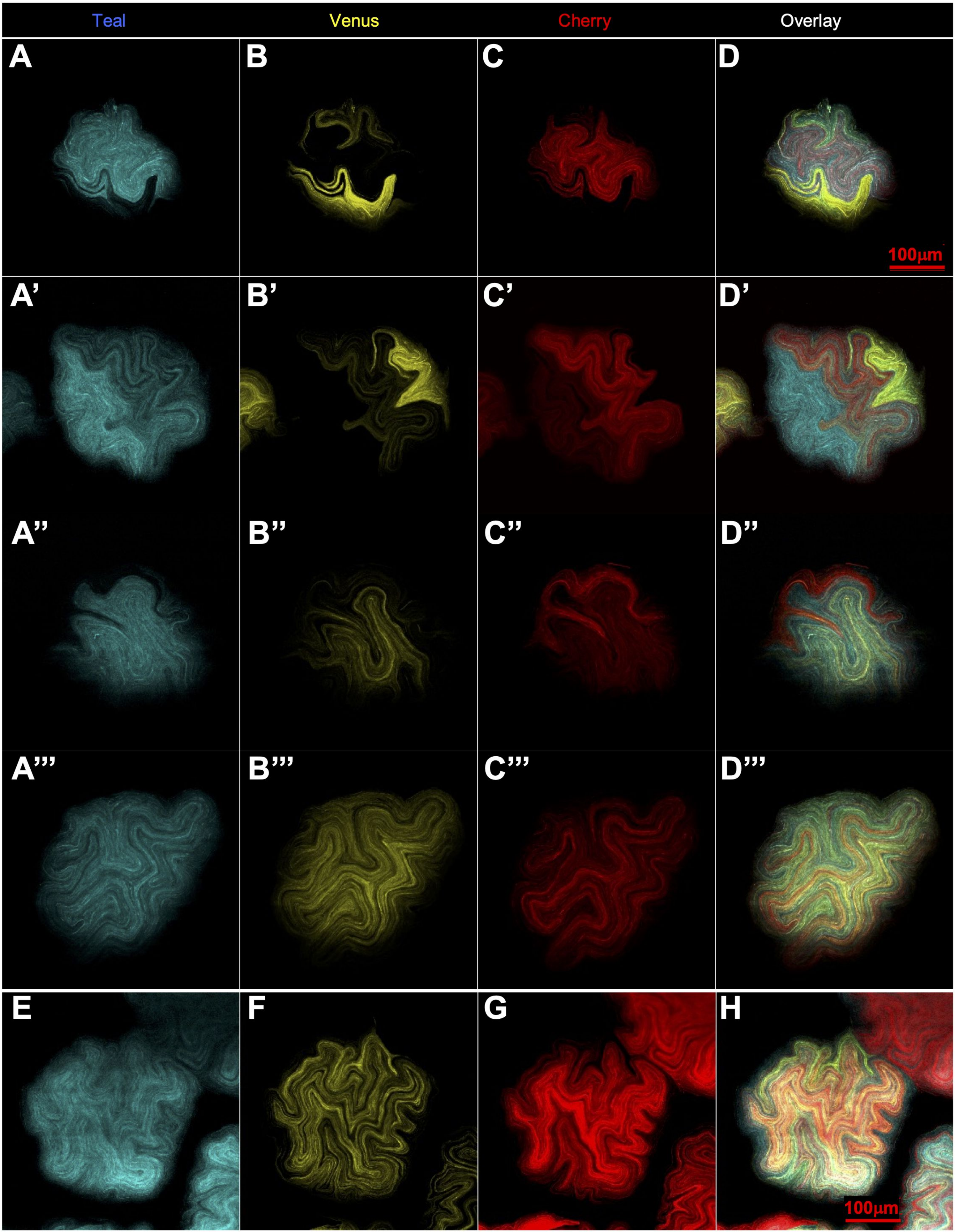

Figure S7

20minute timecourse for mixture colony #1

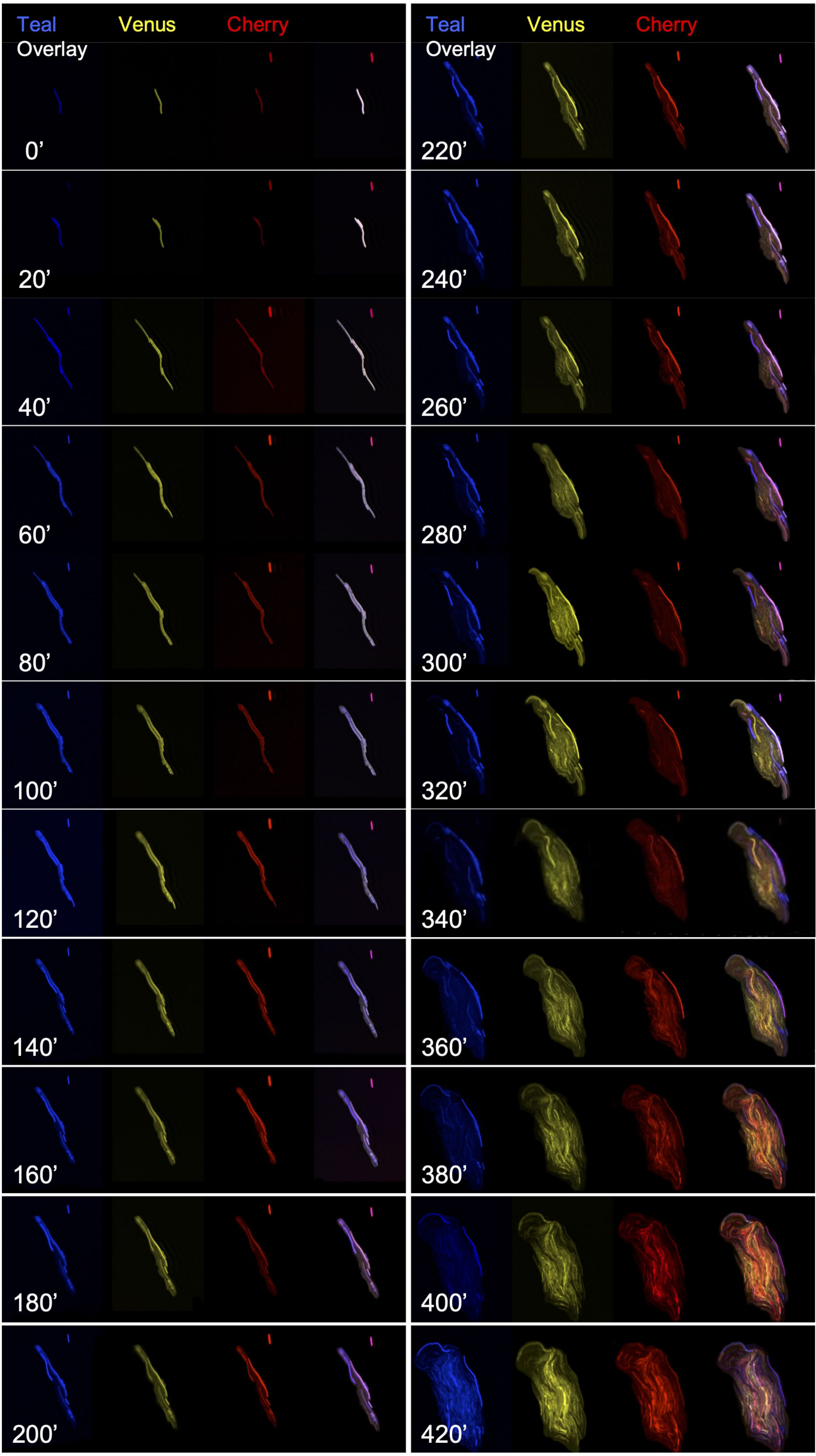

Figure S8

20minute timecourse for mixture colony #2

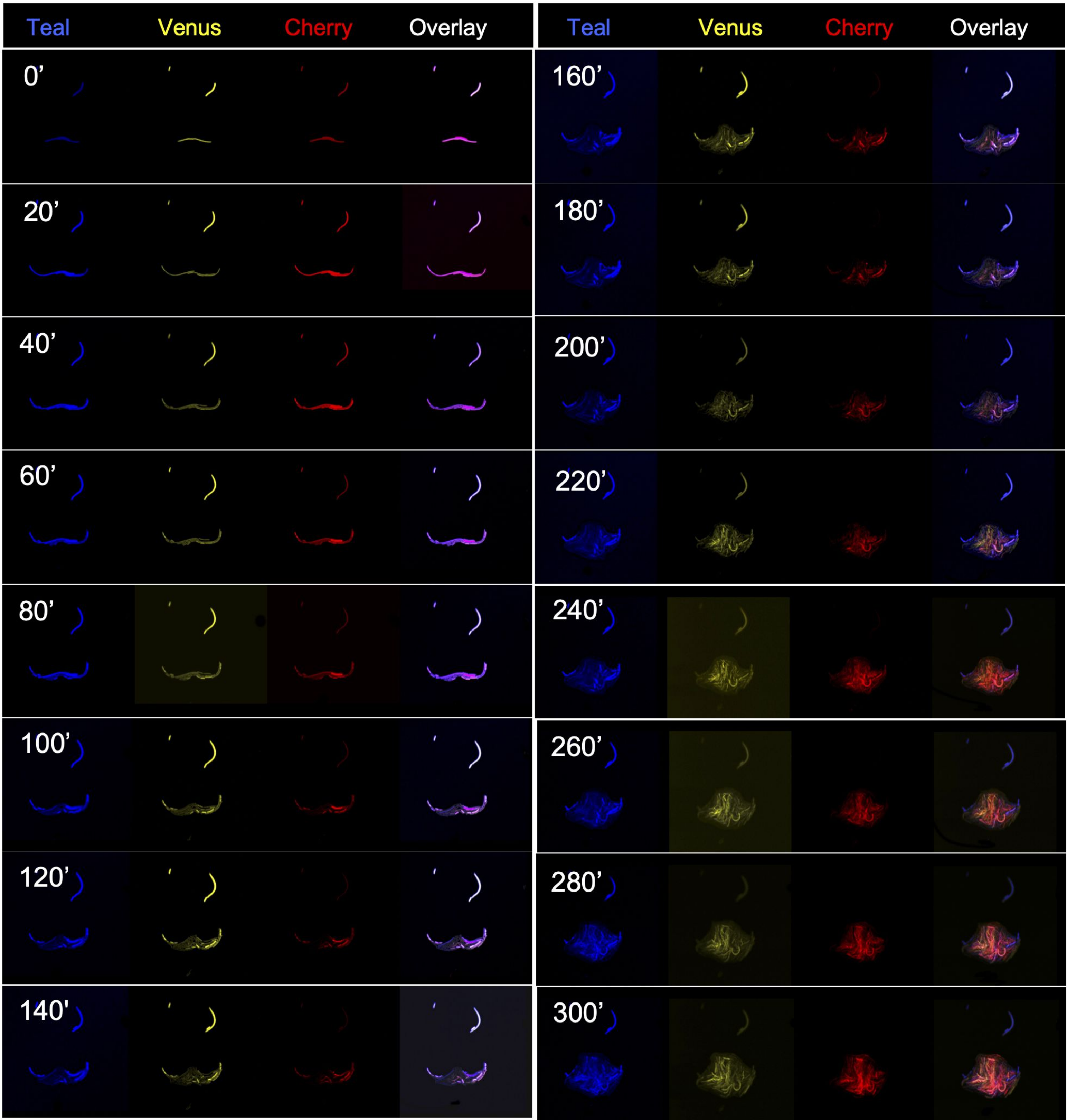

Figure S9

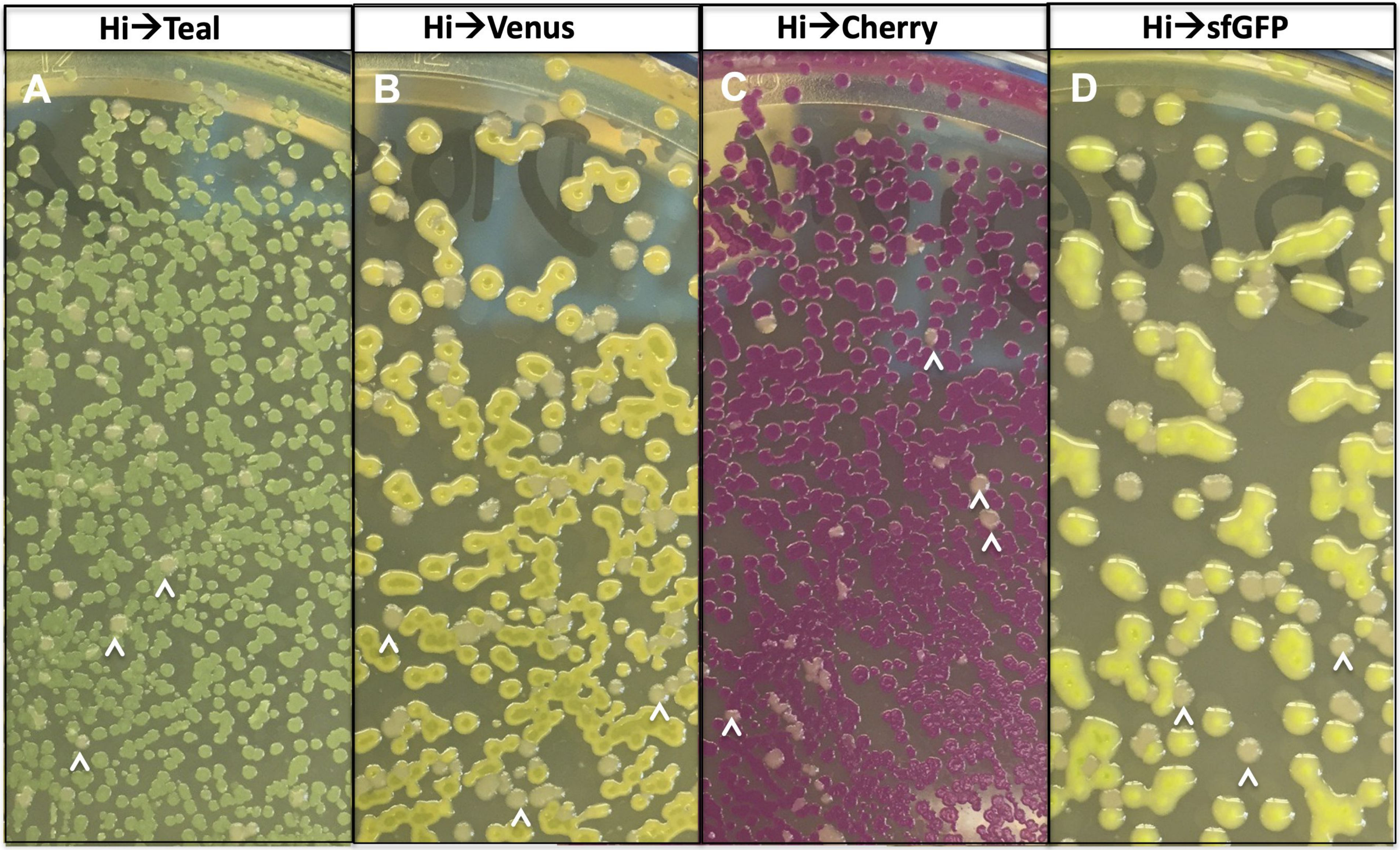

GTCGACCTGCAGGCGGCCGCGAATTCACTAGTGATTTAATTAACGATCCACCGGTCGCCACCATG-XFP

SalI PstI NotI EcoRI SpeI PacI AgeI NcoI

Cloning into Hi-XFP multiple cloning site

Transformation parameters used						
Heat shock	1 min HS			1 min HS		
Conditions	No Wash			Chilled Cuvettes	Chilled Cuvettes	Cold Cuvettes
DH5alpha	PF-Lab			NEB	PF-Lab	PF-Lab
Plasmids	Mix			Mix		
Method	CaCl2			Electroporation		
**Total DNA**	Carbenicillin	Kanamycin	Kan→Carb	Carbenicillin		
Clonal: Percentage of colonies observed with one plasmid						
0.2ng	98.94		95.24			
0.3ng	96.81					
1ng	94.74	96.35	87.88	97.92	95.31	96.70
2ng			80.73			
3ng	91.07			97.83	93.75	
10ng	77.08	86.46	54.17	98.96	96.61	83.22
30ng	72.06	87.85		93.75	94.00	
300ng					53.96	
ODP: Percentage of colonies observed with two plasmids						
0.2ng	1.06		4.76			
0.3ng	3.19					
1ng	4.21	3.65	12.12	2.08	4.69	2.26
2ng			19.27			
3ng	8.04			2.17	6.25	
10ng	18.75	11.46	39.58	1.04	3.13	14.41
30ng	19.58	9.72		6.25	6.00	
300ng					35.97	
OTP: Percentage of colonies observed with three plasmids						
0.2ng			0.00			
0.3ng	0.00					
1ng	1.05	0.00	0.00	0.00	0.00	1.04
2ng			NA			
3ng	0.89			0.00	0.00	
10ng	4.17	2.08	5.21	0.00	0.26	2.37
30ng	8.36	2.43		0.00	0.00	
300ng					10.07	

Table S1: Observed probabilities

Transformation parameters used						
Heat shock	1 min HS	1 min HS	1 min HS	1 min HS	Short HS	Short HS
Conditions Wash	Cold CaCl2	Cold CaCl2	RT Water	RT Water	RT Water	RT Water
DH5alpha	PF-Lab					
Plasmids	Mix	Sep	Mix	Sep	Mix	Sep
Method	CaCl2					
**Total DNA**	Carbenicillin					
Clonal: Percentage of colonies observed with one plasmid						
30ng	72.92	98.96	89.58	100.00	59.52	96.87
ODP: Percentage of colonies observed with two plasmids						
30ng	26.04	1.04	9.38	0.00	32.14	3.13
OTP: Percentage of colonies observed with three plasmids						
30ng	1.04	0.00	1.04	0.00	8.33	0.00

Table S2: Observed probabilities after treatments

Data File S2

We have the following distribution of colors for plasmids C (Cherry), T (Teal) and V (Venus):

# of types	# of colors observed 1	2	3	Calculated probabilities
1 plasmid	**w11**	0	0	**Q1**
2 plasmids	w21	**w22**	0	**Q2**
3 plasmids	w31	w32	**w33**	**Q3**
Observed Probabilities	**P1**	**P2**	**P3**	1

Probabilities table

In the above table, observed probabilities are **P1**, **P2**, and **P3** (the proportion of plasmid types), but we want to compute unobserved and observed probabilities: **Q1**, **Q2**, and **Q3** . The zeroes appear because, once we know the observed plasmids, the number of types cannot be smaller than the number of colors. The numbers in the margins are the sums of the corresponding rows or columns.

For the values of w11, w21, w22, w31, w32 and w33 the conditional probabilities were surmised as follows:

1) There are three types of w11: C, T and V

2) Given a C, T or V event there is an equal chance that a w21 event or w22 event has occurred:
There are three types of w21 events: CC, TT and VV
There are three types of w22 events: CV, CT, and TV

3a) Given there are CC, TT or VV events then,
-there are three types of w31 events: CCC, TTT and VVV
-there are six types of w32 events: CCT, CCV, TTV, TTC, VVC and VVT
-there are zero w33 events
3b) Given there are VT, VC or CT events then,
-there are zero w31 events
-there are six types of w32 events: VTT, VVT, VCC, VVC, CTT, and CCT
-there are three types of w33 events VTC, VCT or CTV.
Thus there are just as many w33 events as w31 and 4 fold more w32 events than w31 or w33 events.

The equations to compute theoretical single, double and triple positives:

P1 is equal to our observed single positives (putatively clonal), w11
P2 is equal to our observed double positives, w22
P3 is equal to our observed triple positives*#, w33
Q1 will be the estimated single positives (clonal)
Q2 will be the estimated double positives
Q3 will be the estimated triple positives

Therefore:
Q3= w33 (w31) + 4x w33 (w32) + w33 (P3)= 6x P3
Q3= 6 x P3

Q2= w21 (w22) + w22 (P2) = 2 x P2, but we must subtract triple events that look like double events (w32) or -4x P3
Q2=2x P2-4x P3

Q1= w11 (P1), but we must subtract double events that look like single events, w21 (w22=P2), and triple events that look like single events, w31 (w33= P3)
Q1= P1-P2-P3

Q1+ Q2 +Q3=P1 + (2xP2-P2)+ (6x P3-4x P3-P3) = P1 +P2 + P3

***Based on the fold reduction in clonality once a w33 event is observed (high number of unobserved triple events), we surmise that 4 plasmid events: w41, 42, w43, begin to occur concomitantly. Thus w33 events signal the beginning of a precipitous drop in clonality.**

#For Kan→Carb experiments there was a single four plasmid event. This event was not added to P3 in Table 1 or Table S1. If this event is added to the P3 percentage, then Q3=37.49%, Q2=54.17% and Q1=8.34%

Supplementary Figure Legends:

Fig. S1. Mixed colonies are not separable into components upon replating.

A, C-F, Replating of mixture of Cerulean, Venus and Cherry fluorescing colonies at high density still show separation of fluorescence. **A and F** are overlay. **B, G-J**, by contrast, replating of a single mixed Cerulean, Venus and mCherry expressing colony at high density retains the fluorescent coexpression in all resulting colonies. **B and J** are overlay.

Fig. S2. Individual bacterial colonies arrayed for screening of fluorescent coexpression.

A. Single colonies were picked at random after transformations and grown overnight in a 96 well plate with 100 μl of selective media. The following day, 1 μl from each well are arrayed on a bacterial plate and grown for 12 hrs at 37 C followed by growth on the bench for 1-2 days. Hi→Teal (T), Hi→Venus (V), and Hi→Cherry (C) bacterial growths are readily visible. Double and triple coexpressing bacterial growths are also visible (TV, VC, CT, and TVC) and were assigned by confocal microscopy. For the double transformants we ranked the higher fluorescent output first in the annotations (see Fig. S3 and Data File S1).

Fig. S3. Screening of bacterial growths for coexpression by fluorescent microscopy.

A-C, A Hi→Teal (T) and Hi→Cherry (C) coexpressing bacterial growth (TC). **D** is overlay showing complete overlap between Teal and Venus fluorescence. **E-G,** A Hi→Venus (V) and Hi→Cherry (C) coexpressing bacterial growth (VC). **H** is overlay showing complete overlap between Venus and Cherry fluorescence. **I-K,** A Hi→Teal, Hi→Venus, and Hi→Cherry coexpressing bacterial growth (TVC). **L** is overlay showing complete overlap between Teal, Venus and Cherry fluorescence. **M-O,** A Hi→Teal bacterial growth contaminated with Hi→Venus and Hi→Cherry bacteria. **P** is overlay showing contamination having different morphology than the main bacterial content. High magnification within **M-P** reveals the details of bacterial contamination: Hi→Venus and Hi→Cherry appear as holes in Teal fluorescent image and streaks at the border of the bacterial growth.

Fig. S4. Cotransformation of plasmids with two different resistances.

A-C, A Kanamycin selected single colony from transformation with Hi→Teal (*lam*) and Lo→Cherry (*kan*). **D** is overlay mosaicism of Teal fluorescence. **E-G,** A kanamycin selected single colony from transformation with Hi→Venus (*lam*) and Lo→Cherry (*kan*). **H** is overlay showing mosaicism of Venus fluorescence. **I-K,** Kanamycin selected single colonies from transformation with Hi→Venus (*lam*) and Lo→Cherry (*kan*). **J** shows mosaicism of unselected *(lam)* Venus fluorescence in one colony contrasted with the high expression in several other

colonies whereas **K** shows selected *(kan)* Cherry fluorescence in all colonies. **L** is overlay. **M-O,** Carbenicilin selected colonies from a Hi→Venus (*lam*) and Lo→Cherry (*kan*) positive colony. **N** shows all selected *(lam)* colonies with Venus fluorescence and **O** shows mosaic unselected *(kan)* Cherry expression in one colony. **P** is overlay. **Q-S,** A kanamycin selected colony cotransformed with 4 plasmids: Hi→Teal (*lam*), Hi→Venus (*lam*), Hi→Cherry (*lam*) and Lo→Cherry (*kan*). Fluorescence in **S** is from Hi→Cherry (*lam*) and not from Lo→Cherry (*kan*) for two reasons, first the laser excitation intensity was reduced to a level that only detects Hi→Cherry and not Lo→Cherry and second the pattern of mosaicism is consistent with what is observed when three plasmids expressing three different fluorescent proteins under the same selective resistance. **T** is overlay.

Fig. S5. Cotransformation of multiple fluorescent plasmids with or without selection have similar mosaicism.

A-D. triple fluorescent bacterium from a triple fluorescent colony expressing Hi→Teal, Hi→Venus, and Hi→Cherry and Overlay. Scale bar at 2µm**. E.** A carbenicillin resistant triple fluorescent colony (Lam→Teal, Lam→Venus, and Lam→Cherry) shows mosaic fluorescence (Projected Z-Stack). **F.** A carbenicillin resistant triple fluorescent colony (Hi→Teal, Hi→Venus, and Hi→Cherry) shows mosaic fluorescence (Projected Z-Stack) with single bacterium resolution. **F**. A kanamycin resistant triple fluorescent colony (Hi→Teal, Hi→Venus, and Hi→Cherry: not Carb selected) shows mosaic fluorescence (Projected Z-Stack) without single bacterium resolution; Same colony as in **Fig. S4.** panel **T**. Despite kanamycin resistance from a Lo→Cherry (*kan*) plasmid, still reveals strong fluorescence similar to colony in **A. E-F,** 500µm bacterial colony.

Fig. S6. Mosaic fluorescence observed at 10hr and 17hr of growth.

A-D, A'-D', A''-D'', and A'''-D''' from 4 additional triple fluorescent expressing Hi→Teal, Hi→Venus, and Hi→Cherry at 10 hrs of growth and 150 µm in width. **D-D'''** are overlays. **E-G** is from a triple fluorescent colony expressing Hi→Teal, Hi→Venus, and Hi→Cherry at 17 hrs of growth and 200 µm in width. **H** is overlay. All five colonies reveal same mosaicism as found in older colonies.

Fig. S7. Time course of triple fluorescent colony development

0 minutes to 420 minutes. A triple fluorescent bacterium (A) expressing Hi→Teal, Hi→Venus, and Hi→Cherry was imaged every 20 minutes for 420 minutes. See also **Movie S1A and S1B.** Mosaicism is quickly revealed after a few cell divisions.

Fig. S8. Time course of triple fluorescent colony development

0 minutes to 300 minutes. A second triple fluorescent bacterium (B) expressing Hi→Teal, Hi→Venus, and Hi→Cherry was imaged every 20 minutes for 300 minutes. See also **Movie S2.** Mosaicism is quickly revealed after a few cell divisions.

Fig. S9. Colorimetric assay for cloning insert DNA.

A) Hi→Teal, B) Hi→Venus, C) Hi→Cherry and D) Hi→sfGFP colonies observed in white light after overnight growth on agar plates. If a 1kb SpeI DNA fragment (an insert) is cloned into any of these vectors at the SpeI site in the common polylinker, then white colonies emerge on the plate (white arrowheads). PCR screening of white colonies confirms that they indeed carry the insert. Thus, a colorimetric assay can be used without fluorescent light to identify subcloned DNA fragments.

Movie. S1A, S1B. Triple fluorescent bacterium. (S1A). Teal FP (blue), Venus FP (yellow), Cherry FP (red) and Overlay views for a single bacterial rod that grew over time and were typically longer than a typical 1x 2 µm rod. Note that a Hi→Teal and Hi→Cherry coexpressing bacterium in the same field of you did not replicate and would have been engulfed by the growing colony had it been allowed to grow longer. It is not clear if this non-replicating bacterium is dead or unable to replicate on the plate. These "contaminating" bacteria may be what are represented in panel **Fig. S3. P. (S1B) Overlay only for split image in (S1).**

Movie. S2. Triple fluorescent bacterium (B) Teal FP (blue), Venus FP (yellow), Cherry FP (red) and Overlay views for a single bacterial rod that grew over time and were typically longer than a typical 1x 2µm rod. Note that two other triple fluorescent bacterium did not replicate would have been engulfed by the growing colony. It is not clear if these non-replicating bacteria are dead or unable to replicate on the plate. These "contaminating" bacteria may be what are represented in panel **Fig. S3. P.**

Movie. S3. Z-stack projection of a triple fluorescent colony (Hi→Teal, Hi→Venus, and Hi→Cherry) reveals fluorescence in individual colonies in several planes.

Movie. S4. Z-stack projection of a second triple fluorescent colony (Hi→Teal, Hi→Venus, and Hi→Cherry) reveals fluorescence in individual colonies in several planes.

Table Legends:

Table S1. Summary of cotransformation rates with multiple plasmids under different parameters

The Kan→Carb column reflects concentrations for Carb only. In addition to this, 1 ng of Kan plasmid was added during cotransformation experiments.

Table S2. Summary of cotransformation rates with multiple plasmids with wash step added after heat shock.
